# Amplicon-Based Multiregion Genomic Characterization of HIV-1 in a Tertiary-Care Hospital in Mexico: Antiretroviral Resistance Mutations and Subtype Diversity

**DOI:** 10.3390/ijms27125571

**Published:** 2026-06-20

**Authors:** Eduardo García-Moncada, Enoc Mariano Cortés-Malagón, Jesús Alejandro Pineda-Migranas, Montserrat Ruiz Santana, Iliana Alejandra Cortés-Ortíz, José Francisco Escutia Domínguez, Daniel Agustín Bravata-Alcántara, Gustavo Acosta-Altamirano, Saúl David Razo-González, Manuel Alberto Castillo Mendez, Mónica Sierra-Martínez, Juan Carlos Bravata-Alcántara

**Affiliations:** 1 Medical School, National Polytechnic Institute, Mexico City 11340, Mexico; eduardo.garcia.moncada@gmail.com (E.G.-M.); jesuspm23@yahoo.com.mx (J.A.P.-M.); 2 Research Division, Hospital Juárez de México, Mexico City 07760, Mexico; emcortes@cinvestav.mx; 3 Genetics Laboratory, Hospital Nacional Homeopático, Mexico City 06800, Mexico; 4 Unidad de Investigación en Salud, Hospital de Alta Especialidad de Ixtapaluca, Servicios de Salud del Instituto Mexicano del Seguro Social para el Bienestar (IMSS-BIENESTAR), Ixtapaluca 56530, Mexico; monserrat29rs@gmail.com; 5 Laboratorio de Genética y Diagnóstico Molecular, Hospital Juárez de México, Mexico City 07760, Mexico; iliancortes@yahoo.com.mx; 6 Facultad de Estudios Superiores Cuautitlán, Campo 1, Universidad Nacional Autónoma de México (UNAM), Mexico City 54740, Mexico; franciscoakmc1514@gmail.com; 7 National School of Medicine and Homeopathy, National Polytechnic Institute, Av. Guillermo Massieu Helguera 239, Ticomán, Mexico City 07320, Mexico; danielb16@live.com; 8 Hospital General de México, Mexico City 06726, Mexico; gustavo.acostaa@salud.gob.mx; 9 Facultad de Estudios Superiores Zaragoza, Campus 2, Universidad Nacional Autónoma de México (UNAM), Iztapalapa, Mexico City 09230, Mexico; david.razo@zaragoza.unam.mx; 10 Academia de Nutrición, Plantel Cuautepec, Colegio de Ciencias y Humanidades, Universidad Autónoma de la Ciudad de México, La Palma, Mexico City 07160, Mexico; manuel.castillo@uacm.edu.mx

**Keywords:** HIV-1 molecular epidemiology, amplicon-based sequencing, circulating recombinant forms, transmitted drug resistance, BF recombinants, genomic surveillance, DeepChek, Mexico

## Abstract

Human immunodeficiency virus type 1 exhibits extensive genetic diversity, which has important implications for molecular epidemiology, recombinant-pattern assessment, and antiretroviral resistance surveillance. In Mexico, HIV-1 molecular surveillance has historically relied mainly on partial *pol* gene sequencing, limiting the ability to compare lineage assignments across *gag*, *pol*, and *env* regions. We analyzed plasma samples from 40 treatment-naïve adults receiving care at a tertiary-care hospital in Mexico using a commercial amplicon-based multiregion HIV-1 genomic sequencing workflow. DeepChek^®^ was used as the primary workflow for read processing, mutation calling, region-level subtype assignment, and antiretroviral resistance interpretation. Resistance interpretation was restricted to antiretroviral target regions with sufficient coverage, mainly reverse transcriptase, protease, integrase, and capsid, when available. Drug resistance mutations were identified in 6/40 participants (15.0%) when mutation-level resistance findings in RT, PR, and IN were considered; one additional sample showed a capsid inhibitor-nonsusceptible NGS call. NNRTI-associated findings were identified in 2/40 patients (5.0%), whereas NRTI- and PI-associated findings were identified in 1/40 patients (2.5%). Accessory or secondary INSTI-associated substitutions were detected in 2/40 patients (5.0%). Region-level subtype analysis revealed frequent discordant assignments across amplified segments, which is consistent with complex mosaic profiles; however, these findings are interpreted as region-level subtypes and recombinant-pattern assignments rather than continuous whole-genome recombination maps. One sample had insufficient RT/PROT/INT coverage for drug resistance interpretation in the complete DeepChek report and was retained only for regions meeting quality thresholds. These findings support the value of multiregion HIV-1 sequencing for local molecular surveillance while emphasizing the need for transparent region-level coverage reporting, cautious interpretation of recombinant-pattern calls, and transparent repository reporting.

## 1. Introduction

Human immunodeficiency virus type 1 (HIV-1) is characterized by marked genetic diversity, which has contributed to the complexity and persistence of the epidemic. This diversity results from error-prone reverse transcription, rapid viral replication, frequent recombination, and selective pressures from host immune responses and antiretroviral therapy [1]. This diversity has generated multiple genetic lineages with distinct geographic distributions, biological properties, and clinical implications [2].

HIV-1 group M, responsible for the global pandemic, is classified into subtypes, sub-subtypes, circulating recombinant forms (CRFs), and unique recombinant forms (URFs) [3,4]. Recombination occurs when different viral strains coinfect the same cell, producing mosaic genomes that can efficiently disseminate within transmission networks [4]. The continuing identification and formal designation of CRFs in the Los Alamos HIV Sequence Database illustrates ongoing HIV-1 recombination and evolution [5].

Latin America shows substantial genetic heterogeneity in HIV-1. While subtype B remains common in many countries, non-B subtypes and recombinant forms are being increasingly reported, especially in South America and the Caribbean [6,7]. In Mexico, available molecular epidemiology data have historically relied largely on partial *pol* sequencing [8], which remains valuable for resistance surveillance but does not fully describe lineages across *gag*, *pol*, and *env* regions [8,9].

Next-generation sequencing can improve regional HIV-1 surveillance by enabling more detailed characterization of HIV-1 genetic diversity, subtype assignment, and resistance-related variation [9,10,11]. However, the interpretation of data generated by commercial amplicon-based assays requires precise terminology and should be anchored to the specific workflow and assay characteristics [12,13,14]. Amplicon-based multiregion sequencing provides broader coverage than single-region *pol* analysis does, but it does not necessarily generate a continuous near-full-length consensus genome. Therefore, subtype and recombinant-pattern assignments should be interpreted according to the regions successfully amplified and covered [9,12,13,14].

The objective of this study was to characterize baseline HIV-1 drug resistance-associated findings and region-level genetic diversity in treatment-naïve adults from a tertiary-care hospital in Mexico using an amplicon-based multiregion HIV-1 sequencing approach. We also report region-level coverage and clarify the limitations of interpreting recombinant patterns from discrete amplified genomic segments.

## 2. Results

### 2.1. Clinical and Virological Characteristics

This study included 40 treatment-naïve adults. Their mean age was 36.1 years, with a median of 34.5 years (IQR: 30.5–41.5). Most participants were male (32 out of 40, or 80%). The median plasma viral load was 780,000 copies/mL (IQR: 600,000–1,800,000), corresponding to 5.89 log_10_ copies/mL (IQR: 5.78–6.26). The median plasma viral load did not differ significantly between patients with and without mutation-level resistance-associated findings.

There were also no statistically significant differences according to age, sex, viral load, or patient-level recombinant category (Table 1). Given the small sample size and the low number of participants with resistance-associated findings, the *p* values were interpreted as exploratory and not as evidence of an absence of association.

### 2.2. Sequencing Quality, Regional Coverage, and Interpretability

All samples were analyzed with the DeepChek workflow using the software-defined minimum coverage threshold of >100 reads for reported covered regions. Region-level coverage was extracted from the complete DeepChek reports and is summarized in Supplementary Table S2. P17, P24, P7, protease, reverse transcriptase, integrase, GP120, and GP41 were evaluated across samples when they met the software coverage threshold. Coverage was not uniform across all regions, with GP120 the most frequently fragmented. This is reported explicitly because region-level coverage determines which subtype assignments and resistance interpretations can be considered reliable.

One important interpretability issue was identified. The complete DeepChek report for Study ID S038 revealed insufficient RT, PROT, and INT coverage for drug resistance interpretation in those regions; therefore, S038 was treated as not interpretable for classical RT/PI/INSTI resistance.

### 2.3. Interpretation of Drug Resistance by Antiretroviral Target Region

When mutation-level interpretation focused on antiretroviral target regions with sufficient coverage was used, 6/40 patients (15.0%; exact 95% CI, 5.7–29.8%) harbored at least one resistance-associated or nonsusceptibility finding. NNRTI-associated findings were identified in 2/40 patients (5.0%; exact 95% CI, 0.6–16.9%), whereas NRTI- and PI-associated findings were detected in 1/40 patients (2.5%; exact 95% CI, 0.1–13.2%). INSTI-associated accessory or low-level findings were identified in 2/40 patients (5.0%; exact 95% CI, 0.6–16.9%). A single capsid inhibitor-related nonsusceptibility call involving lenacapavir was observed in the DeepChek/HIVDb output; this was retained as a descriptive finding and was not interpreted as population-level evidence of capsid inhibitor resistance. This capsid result is reported descriptively and should be interpreted cautiously because it was not part of the original canonical RT/PR/IN resistance endpoint (Table 2).

Resistance interpretation was not presented as genome-wide resistance. Instead, it was restricted to regions with known antiretroviral target relevance and adequate coverage: reverse transcriptase for NRTIs/NNRTIs, protease for PIs, integrase for INSTIs, and capsid when the software/algorithm provided a lenacapavir-related interpretation. Consensus reports, when available, were reviewed using multiple interpretation systems, including GRADE 02/2025, ANRS 35_04/2024, HIVDB v9.8, and Rega v10.0.0 (Supplementary Table S1).

### 2.4. Mutation-Level Resistance Profile

Seven distinct resistance-associated or algorithm-flagged mutations were retained for mutation-level reporting. The clearest classical resistance findings were G190E in reverse transcriptase, associated with NNRTI resistance, and I54T in protease, associated with PI resistance. E138G was interpreted in the context of NNRTI use, particularly rilpivirine. T215N/S was interpreted as a thymidine analog mutation revertant rather than as a classical high-level NRTI resistance mutation. S147G and G163K were treated cautiously as integrase-associated accessory or low-level findings, which is consistent with the need to distinguish major mutations from polymorphic or accessory substitutions. Although each mutation occurred at a low frequency, 6 of 40 patients (15.0%) had at least one resistance-associated finding (Table 3), supporting the presence of baseline resistance-related variants in this cohort of treatment-naïve patients.

### 2.5. Region-Level Subtype Diversity and Regional Mosaic Patterns

**Figure 1:**
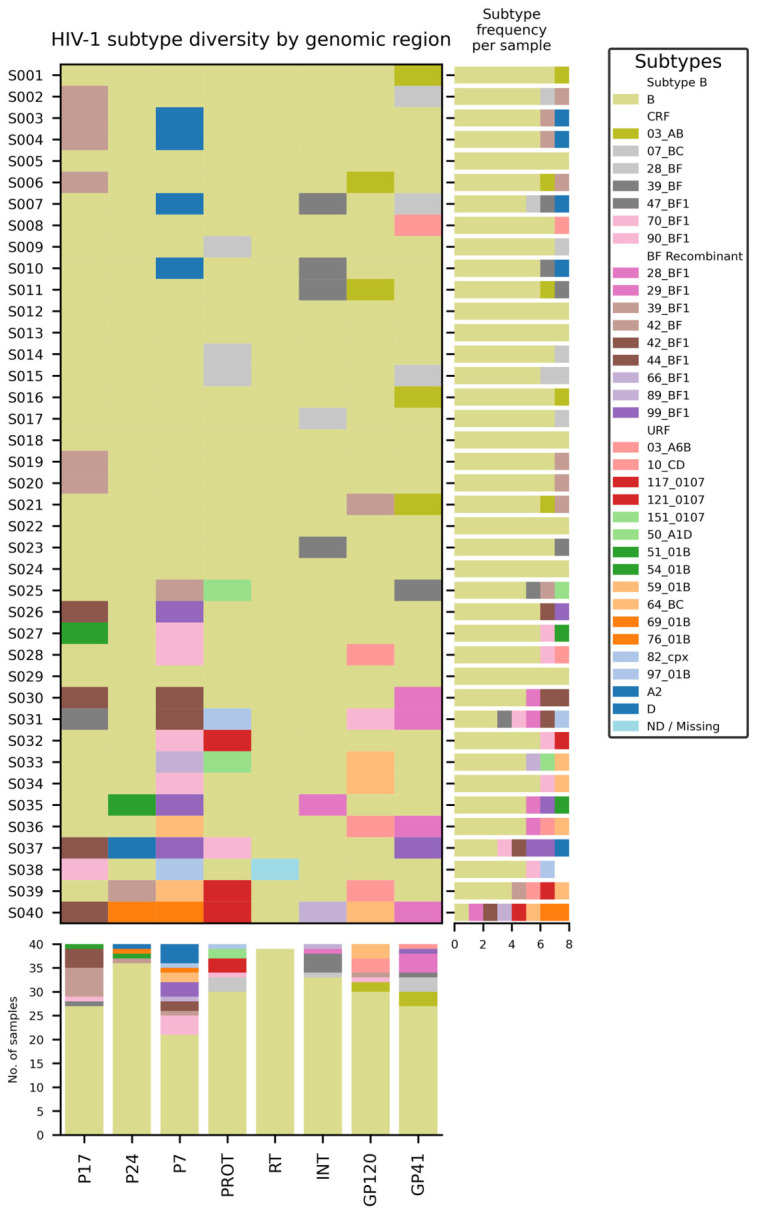
Region-level HIV-1 subtype assignments across eight genomic segments in 40 treatment-naïve patients from Mexico. Heatmap showing subtype or recombinant-lineage assignments across the *gag* (P17, P24, P7), *pol* (PROT, RT, INT), and *env* (GP120, GP41) regions. Each row represents one sample, and each column represents one genomic region analyzed with the DeepChek workflow. Subtype B was the most frequent regional assignment across most segments, whereas non-B, BF-related, CRF-like and URF-like signals were heterogeneous across selected genomic regions. Discordant assignments across regions within the same sample were interpreted as regional mosaic profiles. These patterns should not be interpreted as breakpoint-resolved recombinant structures because independent phylogenetic reconstruction and recombination breakpoint mapping were not performed. The right stacked bars summarize subtype assignments per sample, and the lower stacked bars summarize subtype assignment frequencies per genomic region. Segment-level frequencies may exceed the number of patients because each sample contributed up to eight genomic regions.

Subtype diversity was evaluated at the regional level across eight segments: P17, P24, P7, PROT, RT, INT, GP120, and GP41. The heatmap was retained because it provides a useful visualization of the regional lineage-discordance patterns across samples (Table 4), but its interpretation was revised. It now represents region-level subtype assignments generated for covered amplicon regions, not breakpoint-resolved recombination maps (Figure 1).

The revised interpretation preserves the original biological observation of regional lineage heterogeneity while avoiding overstatement. In the updated heatmap, subtype B is the most frequent regional assignment across most genomic segments; however, non-B, BF-related, CRF-like and URF-like signals are heterogeneously distributed across selected regions and samples. These findings are therefore better described as regional subtype and mosaic profiles inferred from covered genomic segments rather than as definitive breakpoint-defined recombinant structures.

CRF-like and BF-related recombinant-pattern signals were identified across selected genomic regions, including CRF03_AB, CRF07_BC, CRF28_BF and CRF39_BF. In the updated heatmap, these signals are best interpreted as heterogeneous regional assignments superimposed on a largely subtype B background rather than as evidence that recombinant forms predominate across all genomic segments.

Other recombinant patterns involving subtypes C, D, and A were also identified, including BC, CD, A1D, A6B profiles, and complex recombinant forms (cpx). These findings reflect the cocirculation of multiple HIV-1 lineages and the dynamic nature of viral evolution in this population.

A substantial number of URF-like regional mosaic patterns involving multiple subtype fragments were detected. The presence of CRF-like and URF-like regional signals supports the presence of regional mosaic diversity compatible with ongoing recombination; however, these findings should not be interpreted as definitive breakpoint-resolved recombinant structures.

A detailed classification of the recombinant-pattern signals is presented in Table 4. CRF03_AB, CRF28_BF and CRF39_BF were among the recurrent region-level CRF-like assignments, and BF-derived signals were observed in several covered segments. However, because the updated heatmap shows subtype B as the most frequent regional assignment across most segments, these recombinant-pattern signals are interpreted descriptively as regional mosaic/lineage-discordance signals rather than as dominant breakpoint-resolved recombinant genomes. Notably, the frequencies reported for recombinant-pattern signals reflect their occurrence across genomic regions and may exceed the number of patients because multiple regional signals can be detected within individual genomes.

When the data were stratified by recombinant category, DRMs, including subtype B, CRFs, BF recombinants, and URFs, were detected across all the groups (Table 5).

The frequencies in Table 4 represent segment-level recombinant detections and therefore may exceed the number of patients, whereas Table 5 reflects the mutually exclusive patient-level classification used for association analyses.

## 3. Discussion

The present study describes the baseline HIV-1 resistance-associated findings and regional subtype diversity of treatment-naïve individuals receiving care at a tertiary-care hospital in Mexico. The main contribution is not the claim of continuous recombination breakpoint mapping but rather the combined description of antiretroviral target-region resistance, transparent region-level coverage, and regional lineage-discordance patterns across multiple HIV-1 genomic segments.

The resistance findings were uncommon but clinically relevant. The overall proportion of participants with mutation-level resistance-associated findings was 15.0% (95% CI, 5.7–29.8%). Because of the single-center design, small sample size, and high viral load inclusion criterion, this value should not be interpreted as a population-level estimate of pretreatment drug resistance in Mexico. Selection of samples with HIV-1 RNA > 100,000 copies/mL may have favored successful sequencing and may limit generalizability to patients with lower viral loads. Nevertheless, baseline resistance-associated variants are detectable in untreated patients and support the value of baseline genotypic surveillance in tertiary-care settings.

NNRTI-associated findings remained the most frequent classical resistance category in this cohort. These findings are consistent with previous reports documenting pretreatment NNRTI resistance and transmission dynamics in Mexico City [15]. In the contemporary Mexican context, where second-generation integrase inhibitor-based regimens have been implemented nationally [16], baseline resistance testing remains useful not because every accessory mutation changes initial therapy but because it documents the genetic background in which current and future treatment strategies operate.

Among the detected mutations, G190E and I54T were the clearest findings with potential clinical relevance. G190E is a major NNRTI resistance mutation and has been associated with reduced susceptibility to first-generation NNRTIs such as nevirapine and efavirenz [17,18,19]. I54T, detected in the protease region, is also considered a major PI-associated mutation in genotypic resistance interpretation frameworks [19,20]. In contrast, V179E, S147G, and G163K should be interpreted more carefully. These substitutions are generally regarded as accessory or secondary findings, and their individual clinical impact is likely to be limited unless they occur with other resistance-associated changes [18,19,20]. This distinction is aligned with the 2025 IAS-USA update, which emphasizes that mutations vary in their effect and should be interpreted according to drug class, mutational context, and known resistance pathways [19]. Accessory substitutions should therefore be interpreted primarily as markers of viral genetic background rather than as direct predictors of treatment failure.

The RT mutation E138G is classified as an NNRTI-associated mutation linked to reduced rilpivirine susceptibility [19,20]. This is important because it avoids misclassifying the mutation as NRTI related and places it in the correct therapeutic context. T215N/S was interpreted as a thymidine analog mutation revertant rather than as a classical high-level NRTI resistance mutation [19,20,21]. Although revertant mutations do not necessarily confer the same level of resistance as classical thymidine analog mutations do, they may indicate prior evolutionary pathways related to NRTI resistance and can be useful for understanding viral history and transmission dynamics [21]. Taken together, these findings support the value of baseline genotypic resistance assessment, but they also reveal why major mutations and accessory substitutions should be distinguished.

The regional subtype profile was among the most relevant findings of the study. Subtype B was the most frequent assignment across most genomic segments in the updated heatmap, but BF-related, CRF-like and URF-like signals were observed heterogeneously across selected samples and regions. This pattern supports the use of multiregion genomic approaches for HIV-1 molecular epidemiology, particularly in settings where regional lineage and mosaic profiles may be missed by partial *pol*-based analysis [4,5,9]. Importantly, non-B-subtype fragments and URF-like regional mosaic patterns were distinguished from definitive breakpoint-resolved recombinant genomes, emphasizing that not all discordant segmental subtype assignments necessarily represent established recombinant forms. This distinction refines the interpretation of mosaic signals and highlights the complexity captured by region-level multiregion analyses. Partial *pol*-based approaches remain useful for resistance surveillance, but they can miss discordant subtype assignments across genomic regions and may underestimate regional lineage heterogeneity [8,9,14].

These findings are relevant in the Mexican context. Pretreatment resistance to NNRTIs has been documented previously in Mexico, and national treatment strategies have progressively shifted toward second-generation INSTI-based regimens [15,16]. In that therapeutic landscape, baseline resistance testing and genomic surveillance remain useful. Their value is not that every accessory mutation should change treatment selection but that they help place resistance findings and viral diversity within a broader epidemiological framework.

The revised approach strengthens the study without disconnecting it from the original manuscript. The original biological signal—substantial subtype heterogeneity across regions—remains visible, but the claims now match the resolution of the data. This is particularly relevant for env-derived signals, where GP120 coverage is frequently fragmented. Reporting these coverage patterns in Supplementary Table S2 allows readers and reviewers to evaluate which regional calls are well supported and which should be treated cautiously. In addition, the software-defined > 100-read threshold should be understood as an operational coverage threshold rather than evidence of uniformly high sequencing depth across all segments; lineage and variant signals in lower-depth or fragmented regions were therefore interpreted with caution and only in the context of the reported region-level coverage.

DeepChek was retained as the primary commercial workflow because it provides integrated standardized output for read processing, mutation calling, region-level subtype assignment, tropism prediction, and resistance interpretation [12,13]. The complete reports documented the sequencing platform, mapped-read percentage, region-level coverage, software version, expert system, algorithm version, and HIVDb version. The consensus reports further supported cross-algorithm resistance interpretation using GRADE, ANRS, HIVDB, and Rega when available [18,20,22,23,24]. This does not mean that every routine clinical run requires external comparison; rather, in the context of manuscript revision, these outputs were described transparently to improve reproducibility and to avoid the perception of a closed black-box interpretation. For subtype and recombinant-pattern assignment, however, no independent subtyping tool was applied; these results therefore remain pipeline-derived, descriptive region-level signals and not independently confirmed recombinant structures.

Repository information reflects the confirmed NCBI BioProject accession and the assigned BioSample and SRA records. The sequencing data have been submitted to NCBI under BioProject accession PRJNA1476786, and BioSample and SRA records have been processed for the 40 study samples under SRA submission SUB16246675 (Table S3). According to the NCBI repository structure, BioProject is a project-level record that links data associated with a biological research initiative, whereas BioSample and run-level records are distinct repository objects [25,26,27,28]. Public release of the linked records follows the NCBI release settings, upon publication or on the indicated release date, whichever occurs first.

Finally, the complete report for S038 indicated insufficient RT/PROT/INT coverage for classical resistance interpretation; therefore, this sample was excluded from RT/PR/IN resistance interpretation and retained only for genomic regions that met the software-defined coverage threshold.

## 4. Materials and Methods

### 4.1. Study Design and Population

A cross-sectional study was conducted at the Regional High Specialty Hospital of Ixtapaluca, Mexico, between 2024 and 2025. The study included 40 adult patients with confirmed HIV-1 infection who had not received antiretroviral therapy before sample collection. The study protocol was approved by the institutional ethics and research committees (approval number NR-027-2024), and all participants provided written informed consent in accordance with the Declaration of Helsinki.

### 4.2. Sample Collection and Processing

Peripheral blood samples were collected in EDTA-containing tubes and processed within 2 h of collection. The plasma was separated by centrifugation at 1300× *g* for 10 min at room temperature and stored at −80 °C until analysis. Only samples with a plasma viral load > 100,000 copies/mL were included to ensure sufficient viral RNA for amplicon-based multiregion sequencing.

### 4.3. RNA Extraction and Viral LOAD Quantification

Viral RNA was extracted from 140 µL of plasma using the QIAamp Viral RNA Mini Kit (QIAGEN, Hilden, Germany) following the manufacturer’s instructions. Briefly, plasma samples were lysed with buffer containing carrier RNA and incubated at room temperature for 10 min. Ethanol (96–100%) was added, and the mixture was loaded onto QIAamp Mini spin columns (QIAGEN). After being washed with AW1 and AW2 buffers, the RNA was eluted in 60 µL of AVE buffer and stored at −80 °C until use.

An aliquot of the extracted RNA was used for HIV-1 viral load quantification using the Artus^®^ HI Virus-1 RG RT-PCR Kit (QIAGEN, Hilden, Germany) according to the manufacturer’s instructions. Real-time RT-PCR amplification was performed on a CFX96 Real-Time PCR Detection System (Bio-Rad, Hercules, CA, USA), and viral load values were expressed as copies/mL using the assay-provided calibration standards. The RNA concentration was determined using a Qubit RNA Assay Kit (Thermo Fisher Scientific, Waltham, MA, USA).

### 4.4. Amplicon-Based Multiregion HIV-1 Sequencing

Amplicon-based multiregion HIV-1 sequencing was performed using the DeepChek^®^ Whole Genome HIV-1 Genotyping Assay/DeepChek^®^ workflow (ABL Diagnostics, Luxembourg) according to the manufacturer’s protocol [12,13]. Sequencing libraries were prepared following the kit protocol and sequenced on an Illumina MiniSeq platform (Illumina, San Diego, CA, USA) using a mid-output flow cell (300 cycles) with paired-end reads (2 × 150 bp). The term “amplicon-based multiregion HIV-1 genomic sequencing” was used throughout this manuscript to avoid implying a continuous breakpoint-resolved whole-genome assembly when the analytical interpretation was based on covered amplicon regions [9,10,11,12].

### 4.5. DeepChek Analysis, Coverage Thresholds, and Resistance Interpretation

Raw sequencing data were processed using the DeepChek^®^ v2.0 bioinformatics pipeline (ABL Diagnostics), which includes read quality control, trimming, genome assembly, and consensus sequence generation [12,13]. The complete reports provided region-level coverage, mapped-read percentage, mutation profiles, tropism prediction, and drug resistance interpretation. Regions were interpreted according to the software-defined coverage threshold, reported as >100 reads for covered positions. Mutations of interest in the complete reports were classified using HIVDb v9.5 [18,24], and variants detected at ≥20% within the viral population were considered for downstream mutation-level analysis. This threshold may lead to the underestimation of minority resistance variants present below 20%; therefore, the results of this study should not be interpreted as a comprehensive minority-variant analysis. Although the >100-read threshold was used as the software-defined operational criterion for reporting covered regions, lower-coverage or fragmented segments were interpreted cautiously because reduced read depth may affect confidence in minority-variant detection and lineage assignment.

Resistance interpretation was restricted to antiretroviral target regions with sufficient coverage, mainly reverse transcriptase, protease, integrase, and capsid, when available. Consensus reports were reviewed using multiple interpretation systems, including GRADE 02/2025, ANRS 35_04/2024, HIVDB v9.8, and Rega v10.0.0 when available [18,21,22,23,24]. Concordant and discordant resistance interpretations are summarized in Supplementary Table S1.

### 4.6. Region-Level Subtype Assignment and Heatmap Visualization

Definitive breakpoint-level recombination mapping and independent phylogenetic reconstruction were outside the scope of this analysis and were not claimed. Subtype and recombinant-pattern assignments were generated using the DeepChek^®^ v2.0 workflow and were not independently cross-validated with additional subtyping tools such as REGA, COMET, jpHMM, or RIP; therefore, all lineage and recombinant-pattern assignments derive from a single commercial pipeline applied to non-continuous amplicon coverage and are interpreted descriptively as region-level lineage and mosaic signals rather than as independently confirmed recombinant structures.

### 4.7. Ethical Considerations

All procedures were conducted in accordance with institutional guidelines and international ethical standards. Patient data were anonymized before analysis to ensure confidentiality.

### 4.8. Statistical Analysis

Descriptive statistics were used to summarize demographic, clinical, virological and sequencing-related variables. The plasma viral load values were log10-transformed prior to statistical analysis. Continuous variables were compared using the Mann-Whitney U test because of their nonnormal distribution, whereas categorical variables were analyzed using Fisher’s exact test. Exact 95% confidence intervals for proportions were calculated using the Clopper-Pearson method. Statistical significance was set at *p* < 0.05, but all association analyses were interpreted as exploratory because of the small sample size and low number of resistance-associated events.

## 5. Conclusions

This study documents low-frequency but clinically relevant resistance-associated findings and substantial regional lineage discordance across HIV-1 genomic segments. By reporting region-level coverage, distinguishing segment-level detections from patient-level categories, and avoiding overinterpretation of recombinant-pattern calls as breakpoint-resolved recombinant structures, the revised manuscript provides a more transparent and reproducible interpretation of the data.

## Figures and Tables

**Table 1:** Comparison of demographic and virological characteristics between patients with and without HIV-1 drug-resistance mutations (DRMs).

Variable	DRM (+) (*n* = 6)	DRM (−) (*n* = 34)	*p* Value
Age, years, mean ± SD	36.0 ± 4.4	36.1 ± 7.6	0.955
Age, years, median (IQR)	35.0 (33.3–38.3)	34.5 (30.0–41.8)	0.955
Sex, male, *n* (%)	6 (100%)	26 (76.5%)	0.318
Plasma viral load, copies/mL, median (IQR)	810,000 (705,000–1,275,000)	780,000 (525,000–1,800,000)	0.970
Plasma viral load, log_10_ copies/mL, median (IQR)	5.91 (5.85–6.10)	5.89 (5.72–6.26)	0.970

SD, standard deviation; IQR, Interquartile range. Continuous variables were compared using the Mann-Whitney U test, and categorical variables using Fisher’s exact test. *p* values are exploratory because of the small cohort size and low event count.

**Table 2:** Antiretroviral resistance profile inferred from amplicon-based multiregion HIV-1 sequencing.

Drug Class	Interpretable Samples	Patients with ≥1 Resistance-Associated or Nonsusceptible Finding	Drugs Most Frequently Affected	Interpretation	Exact 95% CI
NRTIs	39/40 *	1/40 (2.5%)	Stavudine/thymidine-analog pathway	Low prevalence; interpret as descriptive baseline finding.	0.1–13.2%
NNRTIs	39/40 *	2/40 (5.0%)	Doravirine, efavirenz, etravirine, nevirapine, rilpivirine	Most frequent classical ARV resistance category in the cohort.	0.6–16.9%
PIs	39/40 *	1/40 (2.5%)	Atazanavir/r, indinavir/r, lopinavir/r, nelfinavir, saquinavir/r, tipranavir/r	Low prevalence; darunavir/r susceptibility preserved in the affected sample.	0.1–13.2%
INSTIs	39/40 *	2/40 (5.0%)	Raltegravir, elvitegravir; selected accessory interpretations	Mostly accessory/low-level findings; no high-level DTG/BIC resistance.	0.6–16.9%
Capsid inhibitors	40/40	1/40 (2.5%)	Lenacapavir	Single nonsusceptible NGS call; interpret cautiously as descriptive.	0.1–13.2%

* Interpretable sample count excludes S038 for RT/PROT/INT interpretation because the complete DeepChek report showed insufficient coverage in those regions. Estimates are shown at the cohort level to maintain comparability with the original results.

**Table 3:** Mutation-level resistance-associated findings identified by amplicon-based multiregion HIV-1 sequencing.

Study ID	Region	Mutation (s)	Drug Class	Interpretation/Comment
S021	RT	G190E	NNRTIs	Major NNRTI resistance-associated mutation.
S021	RT	V179E	NNRTIs	Accessory NNRTI-associated substitution; interpreted in mutational context.
S003	RT	E138G	NNRTIs	Reduced susceptibility signal, mainly in the rilpivirine context.
S009	RT	T215N/S	NRTIs	TAM-revertant pathway; not equivalent to classical high-level TAM resistance alone.
S012	IN	S147G	INSTIs	Accessory/secondary INSTI-associated finding; not interpreted as high-level DTG/BIC resistance alone.
S039	IN	G163K	INSTIs	Low-level/accessory INSTI-associated interpretation; interpret cautiously.
S026	PR	I54T	PIs	Major PI-associated mutation; darunavir/r susceptibility preserved in the report interpretation.
S031	P24/capsid	Q67HQ	Capsid inhibitor	Lenacapavir nonsusceptible NGS call in DeepChek/HIVDb output; descriptive only.

RT, reverse transcriptase; IN, integrase; PR, protease; NRTIs, nucleoside reverse transcriptase inhibitors; NNRTIs, nonnucleoside reverse transcriptase inhibitors; PIs, protease inhibitors; INSTIs, integrase strand transfer inhibitors; DTG, dolutegravir; BIC, bictegravir.

**Table 4:** Segment-level subtype/recombinant-pattern detections.

Category	Region-Level Assignment	Segment-Level Detections, *n*	Percentage of 40 Samples Shown for Orientation	Revised Interpretation
CRFs	CRF03_AB	5	12.5%	Region-level CRF-like assignment; not a patient-level prevalence estimate.
CRFs	CRF07_BC	3	7.5%	Detected in specific segments; interpret as regional lineage signal.
CRFs	CRF28_BF	4	10.0%	Latin American BF-related regional assignment.
CRFs	CRF39_BF	4	10.0%	BF-related regional assignment.
CRFs	CRF47_BF1/CRF70_BF1/CRF90_BF1	9	22.5%	BF1-related regional detections; may coexist within patients.
BF recombinants	42_BF/42_BF1 and other BF/BF1 labels	26 *	-	Frequent BF-related regional recombinant-pattern cluster; not a patient-level prevalence estimate and not dominant across all subtype assignments.
URF-like patterns	03_A6B, 51_01B, 59_01B, 64_BC, 82_cpx and related labels	18 *	-	Descriptive regional mosaic/URF-like assignments.
Non-B subtype fragments	A2, D and other non-B fragments	5 *	-	Subtype fragments embedded in regional mosaic profiles.

* Frequencies represent segment-level detections and may exceed the number of patients because each sample contributed to multiple genomic regions. These are not mutually exclusive patient-level categories.

**Table 5:** Association between patient-level HIV-1 recombinant categories and drug-resistance mutations (DRMs).

Patient-Level Working Category	Total (*n* = 40)	Resistance-Associated Finding (+), *n* (%)	No Resistance-Associated Finding, *n* (%)	Exploratory *p* Value
Subtype B predominant/no regional mosaic signal	7 (17.5%)	1 (14.3%)	6 (85.7%)	-
CRF-like regional profile	12 (30.0%)	2 (16.7%)	10 (83.3%)	-
BF-related predominant regional profile	15 (37.5%)	2 (13.3%)	13 (86.7%)	-
URF-like regional mosaic profile	6 (15.0%)	1 (16.7%)	5 (83.3%)	-
Total	40 (100%)	6 (15.0%)	34 (85.0%)	0.97

Patient-level working categories used for exploratory association with resistance-associated findings. Unlike Table 4, this table uses mutually exclusive patient-level categories and therefore sums to 40 participants. The *p* value is exploratory and should not be overinterpreted because only six participants had resistance-associated findings.

## Data Availability

The original contributions presented in this study are included in the article/Supplementary Material. Further inquiries can be directed to the corresponding authors.

## Supplementary Materials

The following supporting information can be downloaded at: https://www.mdpi.com/article/10.3390/ijms27125571/s1.

## Abbreviations

The following abbreviations are used in this manuscript:

ART	Antiretroviral therapy
BF	Recombinant forms derived from subtypes B and F
CI	Capsid inhibitors
CRFs	Circulating recombinant forms
DRMs	Drug-resistance mutations
*env*	Envelope gene region of HIV-1
*gag*	Group-specific antigen gene region of HIV-1
HIV-1	Human immunodeficiency virus type 1
IQR	Interquartile range
IN	Integrase gene
INSTIs	Integrase strand transfer inhibitors
NRTIs	Nucleoside reverse transcriptase inhibitors
NNRTIs	Nonnucleoside reverse transcriptase inhibitors
NGS	Next-generation sequencing
PI	Protease inhibitors
PR	Protease gene
*pol*	Polymerase gene region of HIV-1
RNA	Ribonucleic acid
RT	Reverse transcriptase gene
SD	Standard deviation
URFs	Unique recombinant forms
