# Supplementary material for: Amplicon-Based Multiregion Genomic Characterization of HIV-1 in a Tertiary-Care Hospital in Mexico: Antiretroviral Resistance Mutations and Subtype Diversity
Source: Int J Mol Sci. 2026 Jun 20;27(12):5571. doi: 10.3390/ijms27125571 (PMC13299566; doi:10.3390/ijms27125571)
Supplement: Supplementary file 1 [file ijms-27-05571-s001.zip › ijms-4328691-supplementary.pdf]

Supplementary Materials

Supplementary Tables

Amplicon-based multiregion genomic characterization of HIV-1 in a tertiary-care hospital in Mexico: antiretroviral resistance mutations and subtype diversity

These supplementary tables are aligned with the manuscript. They include the resistance interpretation summary, mutation-level notes, region-level coverage and interpretability summary, and updated NCBI repository metadata, including the BioProject accession, assigned BioSample accessions for the 40 study samples, and processed SRA records under submission ID SUB16246675.

Supplementary Table S1A. Drug-class resistance interpretation summary.

Drug class	Interpretable samples	Patients with >=1 resistance-associated/non-susceptible finding	Representative affected drugs	Interpretation	Exact 95% CI	Affected samples (descriptive NGS matrix)
NRTIs	39/40	1/40 (2.5%)	Stavudine / thymidine-analogue pathway	Low prevalence; descriptive baseline finding.	0.1-13.2%	S009
NNRTIs	39/40	2/40 (5.0%) in manuscript mutation-level endpoint; broader non-susceptible NGS matrix flags additional algorithm-specific calls	Doravirine, efavirenz, etravirine, nevirapine, rilpivirine	Most frequent classical ARV resistance category; interpret according to mutation-level evidence and algorithmic context.	0.6-16.9% for manuscript endpoint	S003, S009, S016, S021 in broader matrix
PIs	39/40	1/40 (2.5%)	Atazanavir/r, indinavir/r, lopinavir/r, nelfinavir, saquinavir/r, tipranavir/r	Low prevalence; darunavir/r susceptibility preserved in the affected sample.	0.1-13.2%	S026
INSTIs	39/40	2/40 (5.0%) in manuscript mutation-level endpoint; broader non-susceptible NGS matrix flags additional algorithm-specific calls	Elvitegravir, raltegravir; selected accessory interpretations	Mostly accessory/low-level findings; no high-level DTG/BIC resistance.	0.6-16.9% for manuscript endpoint	S011, S012, S018, S039 in broader matrix

Drug class	Interpretable samples	Patients with >=1 resistance-associated/non-susceptible finding	Representative affected drugs	Interpretation	Exact 95% CI	Affected samples (descriptive NGS matrix)
Capsid inhibitor	40/40	1/40 (2.5%)	Lenacapavir	Single non-susceptible NGS call; descriptive only.	0.1-13.2%	S031

Note: The primary manuscript endpoint is the mutation-level resistance-associated finding in canonical RT/PR/IN regions with sufficient coverage. The broader NGS matrix is retained only as a descriptive algorithm-specific output and should not be interpreted as the primary prevalence estimate. Capsid/lenacapavir output is reported descriptively and separately from the canonical RT/PR/IN endpoint.

Supplementary Table S1B. Mutation-level resistance-associated findings and interpretive notes.

Study ID	Region	Mutation(s)	Drug class	Interpretation in revised manuscript	Clinical/methodological note
S021	RT	G190E; V179E	NNRTI	Major NNRTI-associated mutation plus accessory substitution	Strongest NNRTI resistance-associated profile; interpret with algorithm support.
S003	RT	E138G	NNRTI	Reduced susceptibility signal, mainly rilpivirine context	Reported as NNRTI-associated, not NRTI-related.
S009	RT	T215N/S	NRTI	TAM-revertant pathway	Marker of prior resistance pathway; not equivalent to classical TAM high-level resistance alone.
S012	IN	S147G	INSTI	Accessory/secondary INSTI-associated finding	Not interpreted as high-level dolutegravir/bictegravir resistance alone.
S039	IN	G163K	INSTI	Low-level/accessory INSTI-associated interpretation	Algorithm-specific low-level elvitegravir/raltegravir interpretation; interpret cautiously.
S026	PR	I54T	PI	Major PI-associated mutation	Most relevant PI finding; darunavir/r preserved in the report interpretation.

Study ID	Region	Mutation(s)	Drug class	Interpretation in revised manuscript	Clinical/methodological note
S031	P24/capsid	Q67HQ	Capsid inhibitor	Non-susceptible lenacapavir call in DeepChek/HIVDb output	Descriptive only; not generalized as capsid inhibitor resistance prevalence.
S038	RT/PR/IN	Not assessed	NRTI/NNRTI/PI/INSTI	Not interpretable in complete report due to insufficient RT/PROT/INT coverage	Excluded from RT/PR/IN resistance interpretation; retained only for regions meeting quality thresholds.

Note: S038 was excluded from RT/PR/IN resistance interpretation because the complete DeepChek report indicated insufficient coverage in those regions.

Supplementary Table S2. Region-level coverage and interpretability summary.

Study ID	Key coverage/interpretable status	Resistance coverage note
S001-S037	P17, P24, P7, PROT, RT, INT, GP120 and GP41 reviewed according to DeepChek >100-read threshold; GP120/GP41 frequently partial or fragmented.	RT/PR/IN interpretable when coverage thresholds were met; cohort-level coverage and interpretability summary shown here.
S038	P17/P24/P7 present; PROT partial, RT insufficient/absent in resistance range, INT partial; GP120 fragmented; GP41 present.	Excluded from RT/PR/IN drug-resistance interpretation because the complete DeepChek report indicates insufficient coverage for RT/PROT/INT.
S039-S040	Core gag/pol segments largely interpretable; GP120 fragmented; GP41 present.	RT/PR/IN interpretable except where explicitly marked otherwise in the accompanying Excel workbook.

Note: This table summarizes region-level coverage and interpretability patterns across the cohort, including the coverage-limited status of S038. Coverage was interpreted according to the software-defined >100-read threshold; lower-coverage or fragmented regions should be interpreted cautiously because reduced read depth may affect confidence in minority-variant detection and lineage assignment.

Supplementary Table S3. NCBI repository metadata associated with the study.

Study ID	BioProject accession	BioSample accession	SRA submission ID	Repository status
S001	PRJNA1476786	SAMN60935569	SUB16246675	BioSample and SRA records processed; public release follows NCBI release settings.

Study ID	BioProject accession	BioSample accession	SRA submission ID	Repository status
S002	PRJNA1476786	SAMN60935570	SUB16246675	BioSample and SRA records processed; public release follows NCBI release settings.
S003	PRJNA1476786	SAMN60935571	SUB16246675	BioSample and SRA records processed; public release follows NCBI release settings.
S004	PRJNA1476786	SAMN60935572	SUB16246675	BioSample and SRA records processed; public release follows NCBI release settings.
S005	PRJNA1476786	SAMN60935573	SUB16246675	BioSample and SRA records processed; public release follows NCBI release settings.
S006	PRJNA1476786	SAMN60935574	SUB16246675	BioSample and SRA records processed; public release follows NCBI release settings.
S007	PRJNA1476786	SAMN60935575	SUB16246675	BioSample and SRA records processed; public release follows NCBI release settings.
S008	PRJNA1476786	SAMN60935576	SUB16246675	BioSample and SRA records processed; public release follows NCBI release settings.
S009	PRJNA1476786	SAMN60935577	SUB16246675	BioSample and SRA records processed; public release follows NCBI release settings.
S010	PRJNA1476786	SAMN60935578	SUB16246675	BioSample and SRA records processed; public release follows NCBI release settings.
S011	PRJNA1476786	SAMN60935579	SUB16246675	BioSample and SRA records processed; public release follows NCBI release settings.
S012	PRJNA1476786	SAMN60935580	SUB16246675	BioSample and SRA records processed; public release follows NCBI release settings.

**Study ID**	**BioProject accession**	**BioSample accession**	**SRA submission ID**	Repository status
S013	PRJNA1476786	SAMN60935581	SUB16246675	BioSample and SRA records processed; public release follows NCBI release settings.
S014	PRJNA1476786	SAMN60935582	SUB16246675	BioSample and SRA records processed; public release follows NCBI release settings.
S015	PRJNA1476786	SAMN60935583	SUB16246675	BioSample and SRA records processed; public release follows NCBI release settings.
S016	PRJNA1476786	SAMN60935584	SUB16246675	BioSample and SRA records processed; public release follows NCBI release settings.
S017	PRJNA1476786	SAMN60935585	SUB16246675	BioSample and SRA records processed; public release follows NCBI release settings.
S018	PRJNA1476786	SAMN60935586	SUB16246675	BioSample and SRA records processed; public release follows NCBI release settings.
S019	PRJNA1476786	SAMN60935587	SUB16246675	BioSample and SRA records processed; public release follows NCBI release settings.
S020	PRJNA1476786	SAMN60935588	SUB16246675	BioSample and SRA records processed; public release follows NCBI release settings.
S021	PRJNA1476786	SAMN60935589	SUB16246675	BioSample and SRA records processed; public release follows NCBI release settings.
S022	PRJNA1476786	SAMN60935590	SUB16246675	BioSample and SRA records processed; public release follows NCBI release settings.
S023	PRJNA1476786	SAMN60935591	SUB16246675	BioSample and SRA records processed; public release follows NCBI release settings.

Study ID	BioProject accession	BioSample accession	SRA submission ID	Repository status
S024	PRJNA1476786	SAMN60935592	SUB16246675	BioSample and SRA records processed; public release follows NCBI release settings.
S025	PRJNA1476786	SAMN60935593	SUB16246675	BioSample and SRA records processed; public release follows NCBI release settings.
S026	PRJNA1476786	SAMN60935594	SUB16246675	BioSample and SRA records processed; public release follows NCBI release settings.
S027	PRJNA1476786	SAMN60935595	SUB16246675	BioSample and SRA records processed; public release follows NCBI release settings.
S028	PRJNA1476786	SAMN60935596	SUB16246675	BioSample and SRA records processed; public release follows NCBI release settings.
S029	PRJNA1476786	SAMN60935597	SUB16246675	BioSample and SRA records processed; public release follows NCBI release settings.
S030	PRJNA1476786	SAMN60935598	SUB16246675	BioSample and SRA records processed; public release follows NCBI release settings.
S031	PRJNA1476786	SAMN60935599	SUB16246675	BioSample and SRA records processed; public release follows NCBI release settings.
S032	PRJNA1476786	SAMN60935600	SUB16246675	BioSample and SRA records processed; public release follows NCBI release settings.
S033	PRJNA1476786	SAMN60935601	SUB16246675	BioSample and SRA records processed; public release follows NCBI release settings.
S034	PRJNA1476786	SAMN60935602	SUB16246675	BioSample and SRA records processed; public release follows NCBI release settings.

Study ID	BioProject accession	BioSample accession	SRA submission ID	Repository status
S035	PRJNA1476786	SAMN60935603	SUB16246675	BioSample and SRA records processed; public release follows NCBI release settings.
S036	PRJNA1476786	SAMN60935604	SUB16246675	BioSample and SRA records processed; public release follows NCBI release settings.
S037	PRJNA1476786	SAMN60935605	SUB16246675	BioSample and SRA records processed; public release follows NCBI release settings.
S038	PRJNA1476786	SAMN60935606	SUB16246675	BioSample and SRA records processed; public release follows NCBI release settings.
S039	PRJNA1476786	SAMN60935607	SUB16246675	BioSample and SRA records processed; public release follows NCBI release settings.
S040	PRJNA1476786	SAMN60935608	SUB16246675	BioSample and SRA records processed; public release follows NCBI release settings.

Note: BioSample and SRA records were processed by NCBI for the 40 study samples under SRA submission SUB16246675. Public release of the linked records follows the NCBI release settings, upon publication or on the indicated release date, whichever occurs first.
